# Tat-protein disulfide-isomerase A3: a possible candidate for preventing ischemic damage in the spinal cord

**DOI:** 10.1038/cddis.2017.473

**Published:** 2017-10-05

**Authors:** Dae Young Yoo, Su Bin Cho, Hyo Young Jung, Woosuk Kim, Goang-Min Choi, Moo-Ho Won, Dae Won Kim, In Koo Hwang, Soo Young Choi, Seung Myung Moon

**Affiliations:** 1 Department of Anatomy and Cell Biology, College of Veterinary Medicine, and Research Institute for Veterinary Science, Seoul National University, Seoul, South Korea; 2 Department of Biomedical Sciences, and Research Institute for Bioscience and Biotechnology, Hallym University, Chuncheon, South Korea; 3 Departments of Thoracic and Cardiovascular Surgery, Chuncheon Sacred Heart Hospital, College of Medicine, Hallym University, Chuncheon, South Korea; 4 Department of Neurobiology, School of Medicine, Kangwon National University, Chuncheon, South Korea; 5 Department of Biochemistry and Molecular Biology, Research Institute of Oral Sciences, College of Dentistry, Gangneung-Wonju National University, Gangneung, Korea; 6 Department of Neurosurgery, Dongtan Sacred Heart Hospital, College of Medicine, Hallym University, Hwaseong, South Korea

## Abstract

In the present study, we searched for possible candidates that can prevent ischemic damage in the rabbit spinal cord. For this study, we used two-dimensional gel electrophoresis followed by matrix-assisted laser desorption/ionization time-of-flight mass spectrometry, in sham- and ischemia-operated animals. As the level of protein disulfide-isomerase A3 (PDIA3) significantly decreased 3 h after ischemia/reperfusion, we further investigated its possible role against ischemic damage using an *in vitro* spinal cord cell line and *in vivo* spinal cord ischemic model. The administration of Tat-PDIA3 significantly reduced the hydrogen peroxide-induced formation of reactive oxygen species and cell death, based on terminal deoxynucleotidyl transferase-mediated biotinylated dUTP nick end labeling and a colorimetric WST-1 assay. Further, Tat-PDIA3 significantly ameliorated the ischemia-induced deficits in motor function, based on Tarlov’s criteria, 24–72 h after ischemia/reperfusion, as well as the degeneration of motor neurons in the ventral horn 72 h after ischemia/reperfusion. Tat-PDIA3 administration also reduced the ischemia-induced activation of microglia and lipid peroxidation in the motor neurons 72 h after ischemia/reperfusion. PDIA3 also potentially ameliorated the ischemia-induced increase in oxidative markers in serum and decreased the activity of Cu,Zn-superoxide dismutase, Mn-superoxide dismutase, and glutathione peroxidase in spinal cord homogenates, 24 h and 72 h after ischemia/reperfusion. These results suggest that Tat-PDIA3 could be used to protect spinal cord neurons from ischemic damage, due to its modulatory action on the oxidative/anti-oxidative balance. Tat-PDIA3 could be applicable to protects neurons from the ischemic damage induced by thoracoabdominal aorta obstruction.

Spinal cord ischemia and reperfusion injury are devastating complications, which follow surgeries implicating the descending and thoracoabdominal aorta, and have an incidence that ranges from 2.7 to 28%.^1, 2^ As neuronal death induced by an abdominal aorta occlusion can be modeled using segmental blood supply to the spinal cord, this is used in animal models to investigate the mechanism of cell death in spinal cord ischemia.^3^

The transient occlusion of the abdominal aorta underneath the renal artery depletes the glucose and oxygen supply to the spinal cord and causes neuronal degeneration in the dorsal and ventral horn of spinal cord.^3, 4, 5^ Ischemia reperfusion activates a series of processes in the neurons of the spinal cord, including glutamate-mediated excitotoxicity, inflammation, and oxidative stress. Among these, one of most important is the reactive oxygen species (ROS)-induced cellular damage, through lipid peroxidation, protein oxidation, and DNA oxidation, which can finally lead to neuronal death.^6, 7^ In addition, ROS produced from mitochondria regulates the apoptotic pathway via the modulation of cytochrome *c* and apoptosis-inducing factor.^8, 9^ Several approaches to overcome neuronal damage in the spinal cord after ischemia/reperfusion have been attempted.^4, 10, 11, 12^ However, there are few comprehensive reports on the protein profile of spinal cord ischemia and the subsequent targeting of neuroprotective agents against this ischemic damage.

In the present study, therefore, we tried to find possible neuroprotective proteins using 2D-gel electrophoresis (2DE) followed by matrix-assisted laser desorption/ionization time-of-flight mass spectrometry (MALDI-TOF MS) in the spinal cord. Thereafter, we investigated the possibility of using differentially expressed proteins as therapeutic targets in spinal cord ischemia.

## Results

### Identification of PDIA3 as a candidate therapeutic agent for spinal cord ischemia

**Figure 1:**
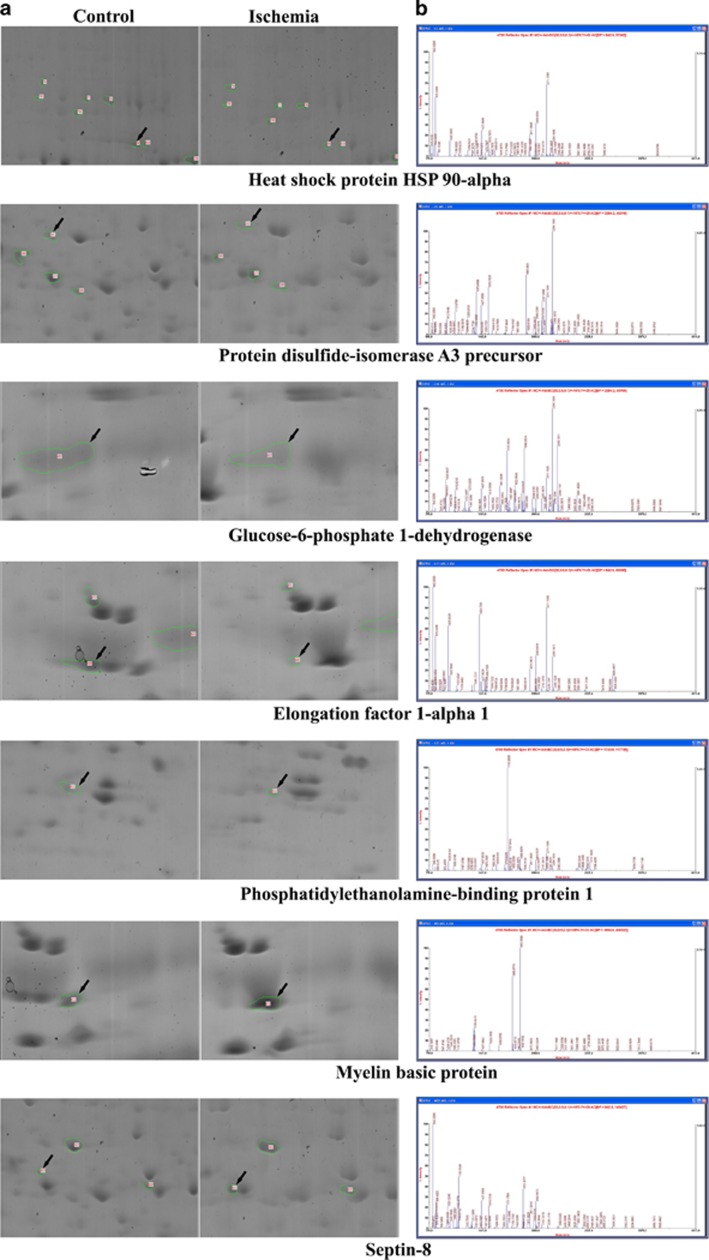
Proteomic approach to find out the possible candidate proteins of spinal cord in the control and ischemia-operated group, 3 h after ischemia/reperfusion. Enlarged view of two-dimensional electrophoresis (2DE) gels. Each panel shows an enlarged view of the 2DE gel spots that were expressed differentially (**a**). The peptide mass fingerprint of the seven candidate spots is also demonstrated (**b**)

To identify therapeutic agents for ischemic damage, we used spinal cord homogenates from the control and 3 h after ischemia, as outlined in Figure 1. On average, 513 and 472 spots were identified in control and ischemic group, respectively, and 398 spots were matched between control and ischemic group (Supplementary Figure 1). Among these spots, seven showed a three-fold increase, compared with the control group, at 3 h after ischemia/reperfusion (Figure 1). In contrast, 28 spots were decreased at 3 h after ischemia/reperfusion, in comparison to the control group. Following the MS analysis of these 35 spots, the predicted proteins were excluded, and we were able to identify seven proteins that were differentially expressed between the control and ischemia samples (Table 1). Among these seven proteins, we chose protein disulfide-isomerase A3 (PDIA3) as a therapeutic candidate protein as it was differentially downregulated, i.e., showed three-fold decrease compared with the control group, in the spinal cord sample 3 h after ischemia.

### Efficiency of *in vitro* Tat-PDIA3 protein transduction in NSC-34 motor neuron-like cells

**Figure 2:**
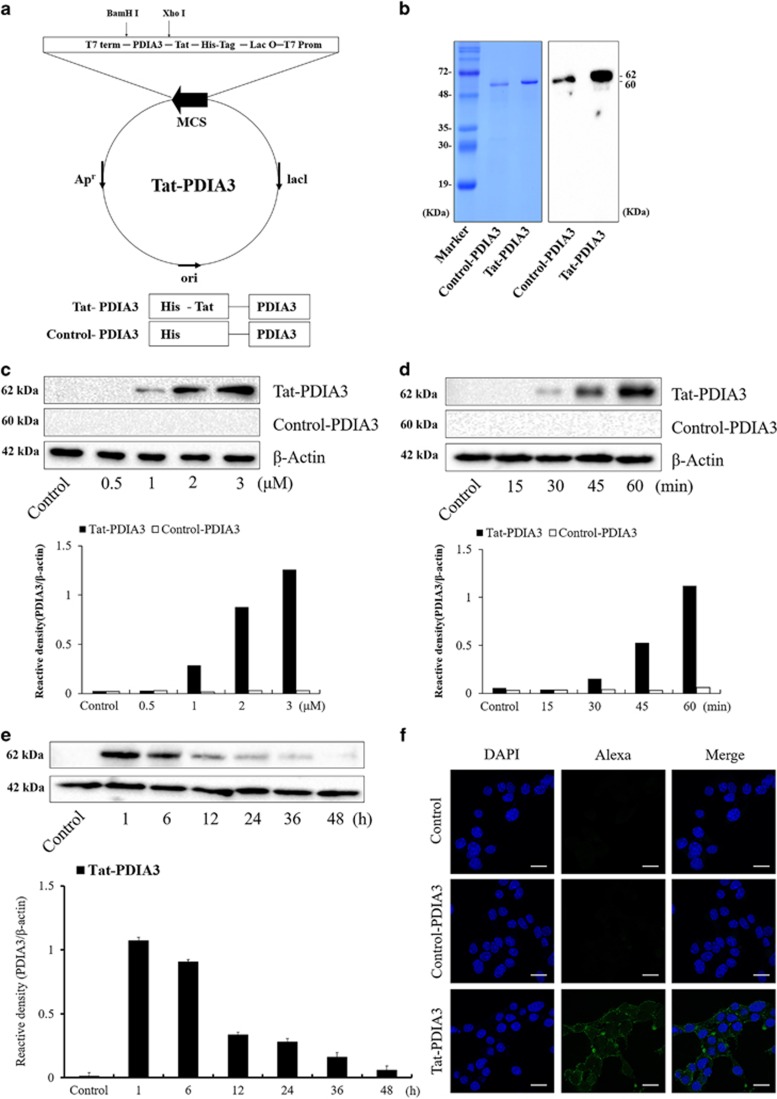
Purification of Tat-protein disulfide-isomerase A3 (Tat-PDIA3) protein and *in vitro* efficiency of Tat-PDIA3 protein transduction into NSC-34 motor neuron-like cells. Overview of Tat-PDIA3 protein (**a**). Expression and purification of control-PDIA3 and Tat-PDIA3 protein, confirmed by polyhistidine western blot analysis (**b**). Time-dependent (0–60 min) transduction of 3 *μ*M treatment with control-PDIA3 or Tat-PDIA3 (**c**). Dose-dependent (0.5–3 *μ*M) transduction of PDIA3 for 1 h after control-PDIA3 and Tat-PDIA3 treatment (**d**). Time-dependent (1–36 h) stability in PDIA3 expression after control-PDIA3 or Tat-PDIA3 protein transduction for 1 h (**e**). Localization of transduced Tat-PDIA3 protein observed with polyhistidine immunocytochemical staining using confocal fluorescence microscopy. Scale bar=20 *μ*m (**f**). The bars indicate mean±S.E.M.

To produce the Tat-PDIA3 fusion proteins, human PDIA3 genes were fused to a Tat peptide expression vector and the control-PDIA3 protein was manufactured without a Tat domain (Figure 2a). Following overexpression, Tat-PDIA3 fusion proteins were purified using a Ni^b+^-→Ni^2+^- nitrilotriacetic acid Sepharose affinity column and PD-10 column chromatography. Western blot analysis, with 15% sodium dodecyl sulfate PAGE (SDS-PAGE) and rabbit anti-polyhistidine antibody, showed strong bands, validating the purified proteins (Figure 2b).

The *in vitro* efficacy of various concentrations of Tat-PDIA3, at different time exposures, was assessed in NSC-34 cells. Tat-PDIA3 fusion proteins transduced efficiently into NSC-34 cells, and transduced PDIA3 was found from 1 *μ*M Tat-PDIA3 treatment, which increased dose dependently 1 h after treatment (Figure 2c). In addition, with the treatment of 3 *μ*M Tat-PDIA3, transduced PDIA3 protein was found from 30 min after treatment and increased time dependent by 1 h after treatment (Figure 2d). However, no transduced PDIA3 expression was found, with any dosage or time exposure, with the treatment of control-PDIA3 (Figures 2c and d). Transduced PDIA3 expression was found in the NSC-34 cells by 48 h after treatment in the NSC-34 cells, although the protein levels decreased in a time-dependent manner (Figure 2e).

Polyhistidine immunohistochemistry with DAPI staining was used to visualize transduced PDIA3 protein expression, 1 h after Tat-PDIA3 treatment. Although polyhistidine immunoreactivity was not found in any structures in the control and control-PDIA3-treated cells, the Tat-PDIA3-treated cells had polyhistidine-positive immunoreactivity in dot-like structures near nucleus and the cell membrane (Figure 2f).

### *In vitro* effects of Tat-PDIA3 protein on H_2_O_2_-induced cell damage in NSC-34 motor neuron-like cells

**Figure 3:**
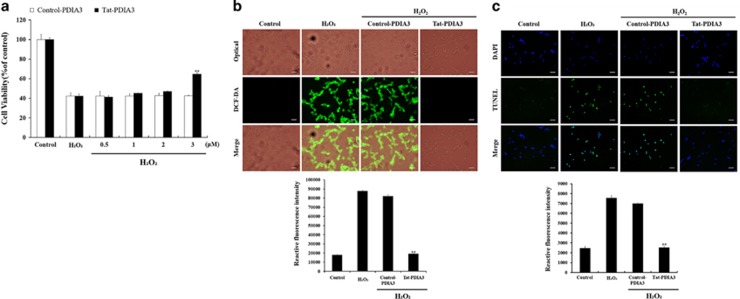
Protective effects of transduced Tat-protein disulfide-isomerase A3 (Tat-PDIA3) protein against 1 mM hydrogen peroxide (H_2_O_2_)-induced reactive oxygen species (ROS) damage in the NSC-34 motor neuron-like cells. Schematic drawing of experimental design (**a**). Cell viability, assessed by WST-1 assay, of NSC-34 cells exposed to H_2_O_2_, at various doses (0.5–3 *μ*M) of control-PDIA3 or Tat-PDIA3 (**b**). H_2_O_2_-induced ROS production, measured by 2′,7′-dichlorofluorescein diacetate (DCF-DA) fluorescence intensity, using an enzyme-linked immunosorbent assay (ELISA) plate reader (**c**). Cell damage based on DNA fragmentation is observed by terminal deoxynucleotidyl transferase-mediated biotinylated dUTP nick end labeling (TUNEL) staining. Scale bar=50 *μ*m (**c**,**d**). Data were analyzed by one-way analysis of variance followed by a Bonferroni’s *post hoc* test, **P<0.01, which was significantly different from the H_2_O_2_-treated group. The bars indicate mean±S.E.M.

Based on the WST-1 assay, exposure to 1 mM H_2_O_2_ caused cell death, with only 40.2–41.8% of NSC-34 cells surviving after a 5 h exposure. Treatment with 0.5–2.0 *μ*M control-PDIA3 or Tat-PDIA3 did not show any significant effects on improving neuronal death in NSC-34 cells. However, treatment with 3.0 *μ*M Tat-PDIA3 showed significantly increased cell survival compared with that in the H_2_O_2_ exposed group, while treatment with 3.0 *μ*M Control-PDIA3 did not show any significant effects on the cell survival (Figure 3a).

ROS formation in the cells was assessed using a 2′,7′-dichlorofluorescein diacetate (DCF-DA)-fluorescent assay after a 5- h exposure to 1 mM H_2_O_2_. NSC-34 cells exposed to 1 mM H_2_O_2_ showed a strong, 4.9-fold increase in DCF-DA fluorescence in comparison to the control group. In comparison to H_2_O_2_-treated cells, DCF-DA fluorescence of Control-PDIA3-treated cells slightly decreased after a 5 h exposure of 1 mM H_2_O_2_. In the Tat-PDIA3-treated cells, the 5- h exposure to 1 mM H_2_O_2_ significantly decreased DCF-DA fluorescence, in comparison to H_2_O_2_-treated cells and was 106% of the untreated control group (Figure 3b).

Terminal deoxynucleotidyl transferase-mediated biotinylated dUTP nick end labeling (TUNEL) staining, was used to visualize neuronal death, was significantly increased by 3.1-fold, in comparison to the control group, after a 5-h exposure of 1 mM H_2_O_2_. Control-PDIA3 treatment and H_2_O_2_-treated cells showed similar TUNEL-positive fluorescence after a 5-h exposure of 1 mM H_2_O_2_. However, Tat-PDIA3 treatment significantly decreased TUNEL-positive fluorescence by 103% of control group (Figure 3c).

### *In vivo* efficacy of Tat-PDIA3 protein against ischemic damage in spinal cord

The administration of vehicle or Tat-PDIA3 did not have any significant effect on physiological parameters such pH, pO_2_, pCO_2_ and glucose levels in the blood or serum, 10 min before and after ischemia/reperfusion (Table 2).

To determine the efficacy and optimal concentration of Tat-PDIA3 protein against ischemic damage in the spinal cord, we measured the neuron survival, based on the Tarlov’s criteria after 24 h and 72 h after ischemia/reperfusion, and neuronal nuclei (NeuN) immunohistochemistry 72 h after ischemia/reperfusion.

**Figure 4:**
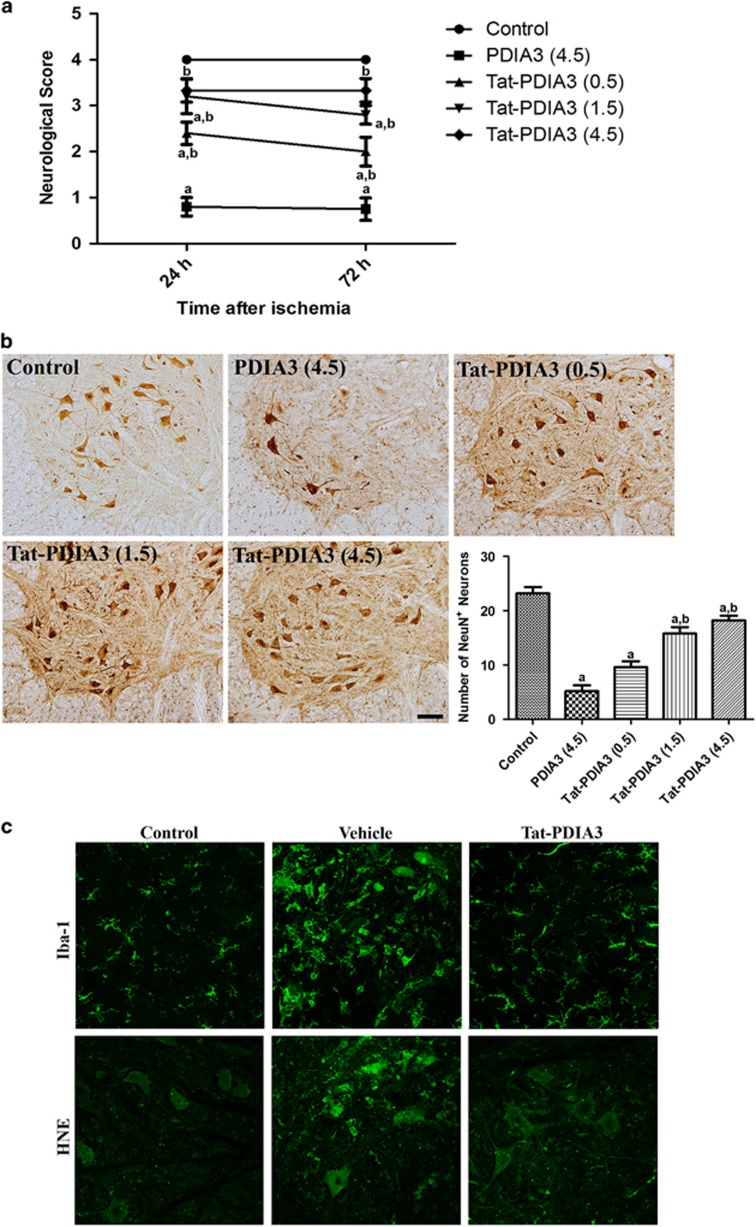
Protective effect of Tat-protein disulfide-isomerase A3 (Tat-PDIA3) protein against ischemic damage in the rabbit spinal cord. Motor behavior assessed using Tarlov’s criteria 24 h and 72 h after ischemia/reperfusion (**a**). Neuronal survival assessed by immunohistochemistry for NeuN in the ventral horn of spinal cord 72 h after ischemia/reperfusion (**b**). Microglial activation and lipid peroxidation assessed by Iba-1 and HNE immunohistochemistry, respectively (**c**). ^a^*P*<0.05 and ^b^*P*<0.05 compared with control, and vehicle-treated group.

In the control group, all animals showed normal behavior, with a neurological score of 4, at 24 h and 72 h after ischemia/reperfusion. In the 4.5 mg/kg PDIA3-treated group, few animals were able to stand and most showed hind-limb abnormality, with an average neurological score of 0.80 and 0.75, at 24 h and 72 h after ischemia/reperfusion, respectively. In the Tat-PDIA3-treated group, many animals were able to stand and move voluntarily, 24 h and 72 h after ischemia/reperfusion in a dose-dependent fashion. However, the administration of 4.5 mg/kg Tat-PDIA3 showed lethality within the 24 h after ischemia/reperfusion in two of the five animals (Figure 4a).

The control group had an abundance of NeuN-positive neurons in the ventral horn of spinal cord, while the 4.5 mg/kg PDIA3-treated group had only a few. The Tat-PDIA3-treated group also had an abundance of NeuN-positive neurons in the ventral horn of spinal cord, which increased in a dose-dependent manner (Figure 4b). Based on the effectiveness and side-effects of Tat-PDIA3, we chose 1.5 mg/kg as the dosage for further experiments to elucidate the possible role of PDIA3 against ischemic damage.

The control group spinal cord had ionized calcium-binding adapter molecule 1 (Iba-1) immunoreactive microglia, with thread-like cytoplasm, and faint 4-hydroxy-2-nonenal (HNE) immunoreactivity. In contrast, the ventral horn of the spinal cord in the vehicle-treated group had Iba-1 immunoreactive microglia with hypertrophied cytoplasm and strong HNE immunoreactivity, 72 h after ischemia/reperfusion. Finally, the ventral horn of the spinal cord in the Tat-PDIA3-treated group only had a few Iba-1 immunoreactive microglia with hypertrophied cytoplasm and weak HNE immunoreactivity 72 h after ischemia/reperfusion (Figure 4c).

### *In vivo* efficacy of Tat-PDIA3 protein against ischemic damage in spinal cord

**Figure 5:**
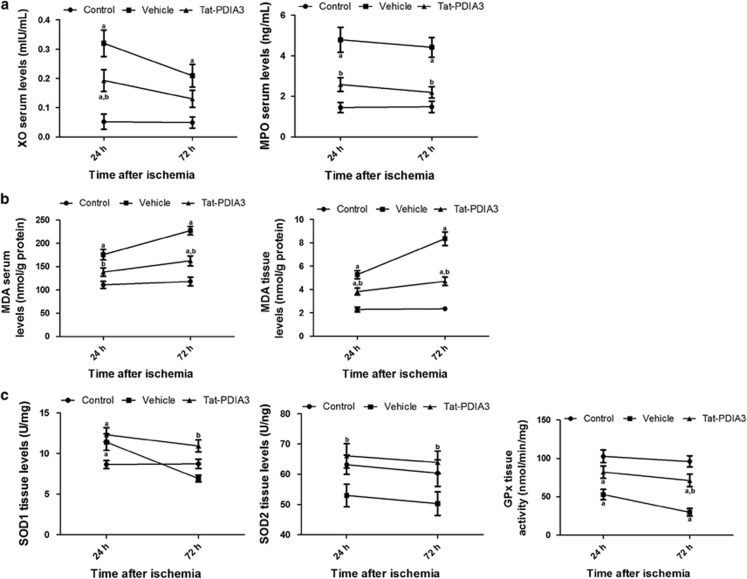
Effects of Tat-protein disulfide-isomerase A3 (Tat-PDIA3) protein on several serum and spinal cord tissue proteins 24 h and 72 h after ischemia/reperfusion. Xanthione peroxidase (XO), myeloperoxidase (MPO) and malondialdehyde (MDA) serum levels were assessed. In addition, levels of MDA, Cu,Zn-superoxide dismutase (SOD1), Mn-superoxide dismutase (SOD2) and glutathione peroxidase (GPx) were measured in tissue samples. ^a^*P*<0.05 and ^b^*P*<0.05 compared with control, and vehicle-treated group.

Compared with the control group, xanthione peroxidase (XO) and myeloperoxidase (MPO) activity in the vehicle-treated group significantly increased, by 6.154- and 3.326-fold, respectively, 24 h after ischemia/reperfusion, and by 4.265- and 2.986-fold, respectively, 72 h after ischemia/reperfusion. In the Tat-PDIA3-treated group, XO and MPO activity significantly decreased by 0.603- and 0.539-fold, respectively, compared with those in the vehicle-treated group 24 h after ischemia/reperfusion. At 72 h after ischemia/reperfusion, XO activity decreased prominently by 0.622-fold although this was not statistically significant (Figure 5a). In contrast, MPO activity decreased significantly by 0.495-fold compared with the control group (Figure 5b).

By 24 h and 72 h after ischemia/reperfusion, the level of malondialdehyde (MDA) in the vehicle-treated group had significantly increased in the serum by 1.586- and 1.922-fold, respectively, and in the tissue by 2.301- and 3.553-fold, respectively, compared with the control group. In addition, the MDA level in the vehicle-treated group further increased 72 h after ischemia/reperfusion, in comparison to 24 h after ischemia/reperfusion. In the Tat-PDIA3-treated group, compared with the vehicle-treated group, at 24 h and 72 h after ischemia/reperfusion, the MDA level significantly decreased in serum (by 0.787- and 0.715-fold) and tissue (by 0.725- and 0.563-fold). The reduced level of MDA was most prominent 72 h after ischemia/reperfusion in both serum and tissue (Figures 5c and d).

Although Cu,Zn-superoxide dismutase (SOD1) activity significantly increased (1.318-fold) 24 h after ischemia/reperfusion, compared with the control, in the vehicle-treated group, there was a sharp drop 72 h after ischemia/reperfusion and SOD1 activity was lower the control group. In the Tat-PDIA3-treated group, SOD1 activity also significantly increased (1.422-fold) 24 h after ischemia/reperfusion, which was maintained at 72 h after ischemia/reperfusion when SOD1 activity was significantly higher (1.579-fold) than the vehicle-treated group (Figure 5e).

Compared with the control, at 24 h and 72 h after ischemia/reperfusion, Mn-superoxide dismutase (SOD2) activity significantly decreased (0.838- and 0.833-fold). In the Tat-PDIA3-treated group, SOD2 activity significantly increased (1.249- and 1.271-fold, respectively) compared with the vehicle-treated group 24 h and 72 h after ischemia/reperfusion, and to the control (Figure 5f).

Compared with the control group 24 h and 72 h after ischemia/reperfusion, glutathione peroxidase (GPx) activity significantly decreased (0.514- and 0.309-fold) in the vehicle-treated group. In the Tat-PDIA3-treated group, GPx activity significantly increased (1.555- and 2.394-fold) 24 h and 72 h after ischemia, compared the vehicle-treated group, although it was lower in comparison to the control group (Figure 5g).

## Discussion

Spinal cord ischemia can result in neuronal damage, inducing immediate or delayed paraplegia as a major complication of surgery for an aorta in the thoracic and abdominal regions. In the present study, we tried to find reliable candidates for neuroprotective or therapeutic agents after spinal cord ischemia in rabbits. Based on 2DE, we found 35 spots that were changed more than 3-fold, compared with control, 3 h after ischemia/reperfusion. We narrowed this list to seven proteins, excluded predicted and unidentifiable proteins, and chose PDIA3 for further analysis since it has thioredoxin-like domains, which showed neuroprotective effects against ischemic damage in the gerbil hippocampus.^13^

PDIA3 facilitates the isomerization of the disulfide bond in nascent and denatured proteins with a thioredoxin-like domain, therefore having an important role in endoplasmic reticulum (ER) stress.^14, 15^ In the present study, PDIA3 protein levels significantly decreased in spinal cord homogenates 3 h after ischemia/reperfusion. This can be attributed to the inactivation of PDI due to the treatment with lipid aldehydes such as MDA, acrolein (ACR), and 4-oxo-2-nonenal (4-ONE)^16^ as PDI promotes the repair of incorrectly formed disulfide bonds. This result is consistent with a previous study demonstrating the significant inhibition of PDI thiol reductase activity 24 h after hypoxia in primary neurons.^17^ In addition, there is enhanced inhibition of PDI via *S*-nitrosylation in Parkinson’s and Alzheimer’s disease brains.^18^ In contrast, however, several lines of evidence demonstrate the increase in PDI in the ischemic or hypoxic brain.^19, 20, 21, 22^ The discrepancy between these results and the current study could be attributed to the different animal models used in the study. We assumed that PDIA3 had been utilized to diminish the accumulation of misfolded or unfolded protein in the ventral horn of spinal cord. However, excessive ROS reduced the level of PDIA3 in spinal cord motor neurons, more than other neurons, due to the higher of unfolded proteins here after spinal cord ischemic injury.^23^ We hypothesized that the supplementation of PDIA3 protects neurons from ischemic damage. To investigate this, we produced Tat-PDIA3 to facilitate the cell penetration of PDIA3 into spinal cord neurons. Tat-PDIA3 efficiently and dose-dependently transduced in NSC-34 motor neuron-like cells and significant levels of transduced PDIA3 levels were observed *in vitro* after 45 min of treatment. In the spinal cord cells, transduced PDIA3 proteins localized to the cell surface and in the cytoplasm, which is supported by a previous study demonstrating PDIA3 distribution over the surface in MC3T3-E1 osteoblasts and in the perinuclear area such as ER.^24^ Our results suggest that Tat-PDIA3 fusion protein efficiently transduced into the cytoplasm without cell surface binding.

Aminopterin-sensitive neuroblastoma N18TG2 with motor neuron-enriched embryonic day 12–14 spinal cord cells form NSC-34 cells, which are motor neuron-like cells.^25^ These cells have action potentials, express neurofilament triplet proteins, and utilize (i.e., synthesize, store and release) acetylcholine.^25^ They have been used to study motor neuron dysfunction in response to toxins,^26^ as well as pathogenic mechanisms in motor neuron disease.^27^ In addition, exposure to H_2_O_2_ caused a significant reduction in NSC-34D cell viability.^28^ In the present study, treatment with Tat-PDIA3 significantly reduced ROS formation and subsequent neuronal death in H_2_O_2_-exposed NSC-34 cells. This result is consistent with the results of the previous studies showing that the overexpression of PDI protected neurons from anoxia, by the inactivation of proapoptotic proteins, such as caspase 3 and caspase 9, as well as by attenuating ER stress.^17^

The *in vivo* results in the current study also demonstrated that the administration of Tat-PDIA3 improved the neurological scores, according to Tarlov’s criteria,^29^ 24 h and 72 h after ischemia/reperfusion. In addition, Tat-PDIA3 significantly reduced spinal cord cell death, in a dose-dependent manner, 72 h after ischemia/reperfusion. Although the overexpression of PDI significantly reduced the number of TUNEL-positive CA1 neurons after transient forebrain ischemia, there was no change in PDI mRNA expression in the hippocampus.^21^ Hoffstrom and colleagues demonstrated that the inhibition of PDI reduced the toxicity of mutant huntingtin exon 1 and A*β* peptides.^30^

In the present study, we observed ischemia-induced free radical formation based on the XO activity, since ischemia facilitates the conversion of xanthine dehydrogenase to XO, which sequentially increases free radical formation,^31^ and the production of neutrophil-derived MPO.^32^ Inhibition of XO protects neurons from the ischemic injury.^33^ The administration of Tat-PDIA3 significantly decreased the XO and MPO activity in the rabbit serum, compared with those in the vehicle-treated group, which suggests that Tat-PDIA3 decreased ROS and MPO production induced by ischemia/reperfusion. These results concur with the results of previous studies showing the significant increase in the level of serum and tissue MPO 24 h after ischemia in spinal cord.^34^ MDA, which is a marker of lipid peroxidation because it is a reliable indicator of progress in neuronal damage,^35, 36, 37^ was significantly increased. A previous also demonstrated the significant increase of the level of MDA in spinal cord tissue 24 h after ischemia.^4, 34^ Since PDI is susceptible to ischemia-induced lipid aldehydes such as MDA, ACR and 4-ONE,^16^ the significant increase in the MDA level could have inactivated it. The administration of Tat-PDIA3, however, ameliorated the ischemia-induced lipid peroxidation in the serum as well as in spinal cord tissue, 24 h and 72 h after ischemia/reperfusion.

To address the relationship between the PDIA3-induced decrease in MDA and antioxidants in the spinal cord, we also measured the activity of SOD1, SOD2 and GPx in the spinal cord, 24 h and 72 h after ischemia/reperfusion. Among these antioxidants, SOD1 significantly increased 24 h after ischemia/reperfusion, while other antioxidants such as SOD2 and catalase significantly decreased in spinal cord tissue.^34, 38, 39^ The administration of Tat-PDIA3 significantly ameliorated the reduction of SOD1, SOD2 and GPx in the spinal cord homogenates after transient forebrain ischemia.

Collectively, our results demonstrate that PDIA3A, a therapeutic candidate protein from the current proteomic study, protected neurons from the ischemic damage in the spinal cord by ameliorating the ischemia-induced lipid peroxidation and reduction of antioxidant enzymes.

## Materials and methods

### Experimental animals

Male New Zealand white rabbits (1.2–1.5 kg), obtained from the Experimental Animal Center (Cheonan Yonam College, Cheonan, South Korea), were housed under standard conditions with adequate temperature (22 ºC) and humidity (60%) control, a 12-h light/12-h dark cycle, and free access to food and water. The handling and care of the animals conformed to the guidelines established to comply with current international laws and policies (NIH Guide for the Care and Use of Laboratory Animals, NIH Publication No. 85-23, 1985, revised 1996). All of the experiments were conducted with an effort to minimize the number of animals used and the suffering caused by the procedures used in the present study. Supplementary Figure 2 outlines the overall workflow of the current study.

### Induction of transient spinal cord ischemia

The animals were anesthetized with a mixture of 2.5% isoflurane (Baxtor, Deerfield, IL, USA) in 33% oxygen and 67% nitrous oxide. A ventral midline incision was made in the abdomen. The abdominal aorta was isolated underneath the left renal artery, and occluded using a non-traumatic aneurysm clip to spare the principal mesenteric flow to the gut. Thereafter, spinal cord ischemia was induced according to a modified method reported by Kiyoshima *et al.*^40^ After 30 min of occlusion, the aneurysm clip was removed, and reperfusion from the abdominal aorta was observed. The body temperature, which was monitored with a rectal temperature probe (TR-100; Fine Science Tools, Foster City, CA, USA), was maintained under free-regulating or normothermic (38.7±0.3 ºC) conditions, using a thermometric blanket before, during, and after surgery until the animals had completely recovered from anesthesia. Thereafter, the animals were kept in a thermal incubator (Mirae Medical Industry, Seoul, South Korea) to maintain body temperature until they were euthanized. Control animals were subjected to the same surgical procedures except that the abdominal aorta was not occluded.

### Protein preparation for 2DE

For 2DE, control animals (*n*=10) and ischemia-operated animals were anesthetized with 2 g/kg urethane (Sigma-Aldrich, St. Louis, MO, USA), 3 h after ischemia/reperfusion (*n*=10 in each time group), and the L_5_–L_6_ spinal cord tissue was isolated from the lumbar vertebrae. Spinal cords were suspended in the sample buffer, which consisted of 30 mM Tris, 7 M urea, 2 M thiourea, 65 mM dithiothreitol (DTT), 4% 3-[(3-cholamidopropyl)dimethylammonio]-1-propanesulfonate (CHAPS) with 40 *μ*l protease inhibitor (pH 8.5). Suspensions were sonicated five times for 10 s and centrifuged at 45 000 rpm for 45 min. Proteins in the supernatants were quantified using the 2D Quant kit (GE Healthcare, Uppsala, Sweden).

### 2DE analysis

For analysis in the first dimension, 1 mg of protein was electrofocused on immobilized pH gradient strips (pH 3–10 nonlinear), as described by Lee *et al.*,^41^ except that a total of 80 000 volts were applied per hour. For separation in the second dimension, isoelectric focusing was performed on an Ettan IPGphor (GE Healthcare) with 24 cm immobilized pH gradient (IPG) strips (pH 4−7, GE Healthcare). After isoelectric focusing, each strip was immersed in 285 *μ*l of isopropanol, 9.7 ml of equilibrium solution (1.5 M Tris-HCl (pH 8.8), 6 M urea, 50% glycerol, 2% SDS, 30% acrylamide), and 15.8 *μ*l of tributyl phosphine. The equilibrated strips were the transferred to 9−16% SDS-PAGE on an Ettan DALT 12 system (GE Healthcare). The preparative gel was stained with Coomassie brilliant blue G250 dye solution overnight, destained using ultrapure distilled water, and scanned using a GS710 scanning densitometer (Bio-Rad, Hemel Hempstead, UK). The gel images were analyzed with Melanie 7 image analysis software (GE Healthcare). Labeled images were uniformly processed using Adobe Photoshop (version CC2014) software.

### Trypsin digestion

For analysis, 2DE spots of interest were excised from the each gel and transferred into 1.5 ml tubes. Each spot was washed with 100 *μ*l of distilled water; then, 50 *μ*l of a 50 mM NH_4_HCO_3_ (pH 7.8) and acetonitrile (6:4) solution was added and the tube was agitated for 10 min. This process was repeated at least three times until the Coomassie brilliant blue G250 dye disappeared. The supernatant was decanted and the spots were dried in speed vacuum concentrator (LaBoGeneAps, Lynge, Denmark) for 10 min. Then, 100 ng per spot was digested with trypsin (Promega, Southampton, UK) in 50 mM ammonium bicarbonate and left on ice for 45 min. Spots were then incubated at 37 °C for 12 h.

### Protein identification using MALDI-TOF MS

Tryptic peptides were desalted and purified using a mixture of Poros R2 and Oligo R3 (Applied Biosystems, Foster City, CA, USA). The MS spectra of peptides were generated by spectrometric analysis using a 4800 MALDI-TOF analyzer (Applied Biosystems) in the reflectron/delayed extraction mode, with an accelerating voltage of 20 kV. Data were summed from 500 laser pulses. The operating software used was Applied Biosystems 4000 series Data Explorer version 4.4 and the T2D file that was obtained from the 4800 MALDI-TOF was opened in Data Explorer. The peaks were filtered using the following four macro processes: (1) baseline correction (peak width=32, flexibility=0.5, degree=0.1), (2) noise filter/smooth: filter coefficient=0.7, (3) spectrum peak deisotoping: adduct=H, generic formula=C_6_H_5_NO, and (4) mass calibration. The spectrum was calibrated with the reference to tryptic autodigested peaks (m/z 842.5090, 1045.564 and 2211.1046) and monoisotopic peptide masses were obtained with Data Explorer. An 800−4000 m/z mass range was used with 1000 shots per spectrum. At the end of the macro process, raw data were generated about centroid mass, resolution, height and *S/N* ratio of each peak. These data were converted to an Excel file and entered into a MASCOT search.

### Data searches for protein identification

MASCOT (Matrix Science, London, UK; version 2.2.04) was used to identify peptide sequences present in the protein sequence database NCBInr (Rabbit). Database search criteria were as follows: MALDI-TOF: NCBInr_Rabbit_20150410 fixed modification, carboxyamidomethylated at cysteine residues; variable modification, oxidized at methionine residues; maximum allowed missed cleavage, 1; peptide MS tolerance. Only peptides resulting from trypsin digests with protein scores that were greater than 66 were considered.

### Cell preparation

NSC-34 cells, derived from the fusion of mouse neuroblastomas and motor neurons from embryonic mouse spinal cords,^25^ were purchased from Bio-Medical Science Co., Ltd (Seoul, South Korea). NSC-34 cells were maintained in Dulbecco’s modified Eagle’s medium (DMEM) supplemented with 10% fetal bovine serum (FBS) and 1% penicillin–streptomycin (PS), and were subcultured every 3–5 days as shown by Cashman *et al.*^25^ To slow cell proliferation and enhance differentiation, the medium was exchanged for 1:1 DMEM and Ham’s 12 with 1% FBS, 1% PS and 1% non-essential amino acids as described by Eggett *et al.*^42^ The medium was changed every two days and the cells were grown for up to seven days, allowing them to differentiate into motor neuron-like cells with increased neurite formation.^42^

### Construction of expression vectors

A Tat expression vector was prepared as described in the previous study.^43^ To construct a Tat-PDIA3 protein, PDIA3 cDNA was amplified by polymerase chain reaction (PCR) with the use of the following primers: sense primer 5′-CTCGAGATGCGCCTCCGC-3′ and antisense primer 5′-GGATCCTTAGAGATCCTCCTGTGCC-3′. After subcloning the PCR product into a TA cloning vector, it was ligated into the Tat expression vector. A PDIA3 expression vector without the Tat-protein transduction domain was also constructed to be used as a control (Control-PDIA3).

The Tat-PDIA3 and control-PDIA3 plasmids were expressed in *Escherichia coli* BL21 cells, which were treated with 0.1 mM isopropyl-*β*-d-thiogalactoside (IPTG; Duchefa, Haarlem, Netherlands) at 18 °C for 8 h, and purified using a Ni^2+^-nitrilotriacetic acid Sepharose affinity column and PD-10 column chromatography (Amersham, Braunschweig, Germany) according to the manufacturer's instructions. The purified proteins were treated using Detoxi-Gel™ endotoxin removing gel (Pierce, Rockford, IL, USA) to remove endotoxins. Endotoxin levels in the proteins were below the detection limit (<0.1 EU/ml) as tested using a Limulus amoebocyte lysate assay (BioWhittaker, Walkersville, MD, USA). The Bradford assay^44^ was used to estimate the quantity of purified Tat-PDIA3 and control-PDIA3 protein.

### Transduction of Tat-PDI3A proteins into NSC-34 cells

To examine the time and concentration-dependent transduction ability of Tat-PDIA3 protein, NSC-34 cells were exposed to different concentrations of the Tat-PDIA3 and PDIA3 proteins, ranging from 0.5–3 *μ*M, for 1 h, and to 3 *μ*M of Tat-PDIA3 and PDIA3 proteins for various time periods, ranging from 15–60 min. Cells were then washed with PBS and treated with trypsin-ethylenediaminetetraacetic acid (trypsin-EDTA). The amount of transduced proteins was measured by western blotting. The intracellular stability of Tat-PDIA3 protein was also examined after being harvested at various times (1–24 h) using a rabbit anti-polyhistidine antibody (Santa Cruz Biotechnology, Santa Cruz, CA, USA).

### Western blot analysis

Equal amounts of proteins were analyzed using 12% SDS-PAGE. Analyzed proteins were electrotransferred to a Polyvinylidene difluoride membrane and the membrane was blocked with TBS-T (25 mM Tris-HCl, 140 mM NaCl, 0.1% Tween 20, pH 7.5) buffer containing 5% non-fat dry milk. The membrane was analyzed by western blot using primary antibodies recommended by the manufacturer. Proteins were identified using chemiluminescent reagents as recommended by the manufacturer (Amersham, Franklin Lakes, NJ, USA), as described previously.^45^

### Confocal fluorescence microscopy

A confocal fluorescence microscope was used to determine the intracellular distribution of transduced Tat-PDIA3 protein in the NSC-34 cells as described previously.^45^ Culture media were placed on coverslips and treated with 3 *μ*M Tat-PDIA3 protein. After 1 h of incubation at 37 °C, the cells were washed with PBS twice and fixed with 4% paraformaldehyde for 5 min. The cells were treated in PBS containing 3% bovine serum albumin, 0.1% Triton X-100 (PBS-BT) at room temperature for 30 min, and washed with PBS-BT. The primary antibody (His-probe, Santa Cruz Biotechnology) was diluted 1:2000 and incubated at room temperature for 4 h. The secondary antibody (AlexaFluor 488; Invitrogen, Carlsbad, CA, USA) was diluted 1:15 000 and incubated in the dark for 1 h. Nuclei were stained with 1 *μ*g/ml DAPI (Roche Applied Science, Mannheim, Germany) for 2 min. Stained cells were analyzed using a confocal fluorescence microscope confocal laser-scanning system (Bio-Rad MRC-1024ES, 4BIOROD, CA, USA).

### DNA damage and cell viability assay

To examine if the transduced Tat-PDIA3 proteins protect against DNA damage, NSC-34 cells were pre-treated with 3 *μ*M Tat-PDIA3 protein for 1 h and exposed to 1 mM hydrogen peroxide (H_2_O_2_) for 3 h. TUNEL and a Cell Death Detection kit (Roche Applied Science) were used to assess cellular damage. Images were analyzed using a fluorescence microscope (Nikon eclipse 80i, Tokyo, Japan).^43, 45^ Fluorescence levels were measured using a Fluoroskan enzyme-linked immunosorbent assay (ELISA) plate reader (Labsystems Oy) with a 485 nm excitation and 538 nm emission.

The biological activity of Tat-PDIA3 protein was measured by assessing cell viability based on WST-1 assay kit (Daeillab Service, Seoul, South Korea) after exposure to H_2_O_2_ as described previously.^43, 45^ NSC-34 cells were plated at a confluence of 70% in a 96 well plate and exposed to Tat-PDIA3 and PDIA3 proteins (0.5–3 *μ*M). After 1 h, cells were treated with 1 mM H_2_O_2_ for 5 h. Cell viability was measured at 450 nm using an ELISA microplate reader (Labsystems Multiskan MCC/340, Helsinki, Finland) and cell viability was expressed as a percentage of untreated control cells.

### Measurement of intracellular ROS levels

Intracellular ROS levels were measured using DCF-DA, which converts to fluorescent DCF in cells when exposed to ROS as described previously.^43, 45^ ROS levels were measured in NSC-34 cells, in the presence or absence of Tat-PDIA3 protein (0.5–3 *μ*M). After 1 h of pre-treatment with Tat-PDIA3 protein, the cells were treated with H_2_O_2_ (1 mM) for 10 min. The cells were washed with PBS and treated with DCF-DA at a dose of 20 *μ*M for 30 min. Fluorescence levels were measured using a Fluoroskan ELISA plate reader (Labsystems Oy, Helsinki, Finland) at a 485 nm excitation and 538 nm emission.

### Experimental group for PDIA3 concentration determination against ischemic damage *in vivo* experiments

To determine the optimal concentration of Tat-PDIA3 that shows neuroprotective effects against ischemic damage in the spinal cord, the animals were divided into two groups: sham-operated (control) and ischemia group. The ischemia group was further divided into four subgroups: 4.5 mg/kg PDI, 0.5 mg/kg Tat-PDIA3, 1.5 mg/kg Tat-PDIA3 and 4.5 mg/kg PDIA3-treated groups. Tat peptide- and glycerol-treated groups were omitted because Tat peptide- or glycerol did not show any neuroprotective effects against ischemic damage in a previous study.^43^ Transient spinal cord ischemia was induced by occlusion of the abdominal aorta in the subrenal region as described above.

### Physiological monitoring before and after ischemia/reperfusion

In all groups, arterial blood gases (PaO_2_ and PaCO_2_), pH, and glucose were measured using a GEM Premier 3000 gas analyzer (Instrumentation Laboratory, Milan, Italy), before ischemia/reperfusion and 10 min after. In addition, the mean arterial pressure (MAP) was recorded from the caudal artery by use of physiography (Physiograph; Gould Instrument Systems, OH, USA) and acquisition software (Ponemah version 3.0; Gould Instrument Systems).

### Neurological assessment

For the assessment of neurological function, modified Tarlov criteria were used as follows:^29^ 0, no voluntary hind-limb function; 1, only perceptible joint movement; 2, active movement but unable to stand; 3, able to stand but unable to walk; or 4, completely normal hind-limb motor function.^4, 12, 46, 47^ All assessments were performed under blinded conditions to ensure objectivity, with two observers for each experiment. The assessments of control, PDIA3-treated, and Tat-PDIA3-treated groups were conducted under the same conditions. Neurological function was assessed 24 h and 72 h after reperfusion because the neurological function of the animals start to deteriorate ~12–24 h after reperfusion, progressing to complete delayed-onset paraplegia within the ensuing 48 h.^48, 49^

### Immunohistochemistry for NeuN in the spinal cord

For the histological analysis, animals used in the neurological assessment (*n*=5 in each group) were anesthetized with 2 g/kg urethane (Sigma-Aldrich) 72 h after ischemia/reperfusion, and perfused transcardially, first with 0.1 M phosphate-buffered saline (PBS, pH 7.4), followed by 4% paraformaldehyde in 0.1 M phosphate buffer (pH 7.4). The animals’ spinal cords were removed, and the 5–6th lumbar segments (L_5_–L_6_) of the spinal cord were post-fixed in the same fixative for 12 h. The spinal cord tissues were cryoprotected by infiltration with 30% sucrose overnight and 30-*μ*m-thick spinal cord sections were cut serially in the coronal plane using a cryostat (Leica, Wetzlar, Germany).

To ensure that the immunohistochemical data were comparable between groups, sections were carefully processed under similar conditions in parallel. Five sections in each group were sequentially incubated with 0.3% H_2_O_2_ in PBS for 30 min and 10% normal goat serum in 0.05 M PBS for 30 min. Sections were then incubated with a mouse anti-NeuN (1:1000; Chemicon International, Temecula, CA, USA) overnight at room temperature. Sections were then incubated with biotinylated goat anti-mouse IgG followed by a streptavidin–peroxidase complex (1:200, Vector, Burlingame, CA, USA). Immunostaining was visualized by reaction with DAB in 0.1 M Tris-HCl buffer (pH 7.2). Sections were dehydrated and mounted on gelatin-coated slides in Canada balsam (Kanto, Tokyo, Japan).

The measurement of NeuN-positive cells in all the groups was performed using an image analysis system equipped with a computer-based CCD camera (software: Optimas 6.5, CyberMetrics, Scottsdale, AZ, USA), in a tissue area observed under 100 × primary magnification. In brief, NeuN-positive neurons were counted at the center of the ventral horn of the spinal cord. The image was converted to grayscale and NeuN-positive neurons were automatically selected according to the intensity of NeuN immunostaining. Cell counts were obtained for each animal by averaging the counts from five sections in each group, and the cell number was reported as a percentage of that obtained from the control groups.

### Serum and tissue sampling for neuroprotective mechanisms of PDIA3 against ischemic damage *in vivo*

Animals from the control, vehicle-, and Tat-PDI-treated groups (*n*=5 in each group) were used to confirm the effects of Tat-PDI on XO and MPO activity, serum levels of MDA, and MDA, SOD1, SOD2 and GPx in spinal cord tissue. The animals were anesthetized with 2 g/kg urethane (Sigma-Aldrich), 24 h and 72 h after ischemia/reperfusion. Blood was isolated from the right ventricle, centrifuged (10 min, 1500 × *g*, 4 °C), and they were stored in liquid nitrogen until measurement. Spinal cord (L_5_–L_6_ segments) was dissected out and homogenized in 10 mM Tris buffer containing 1 mM EDTA or 1 mM phenylmethanesulfonylfluoride, respectively. The homogenates were centrifuged at 600 × *g* for 10 min, and then centrifuged at 13 000 × *g* for 20 min at 4 °C.

### Serum XO and MPO activity as well as MDA level

Serum XO activity was measured by the method described by Prajda and Weber,^50^ where activity is indirectly measured by the amount of uric acid formed from xanthine. Serum samples (100 *μ*l) were incubated for 30 min at 37 °C in 3 ml of the phosphate buffer (pH 7.5, 50 nM) containing xanthine (4 mM). The reaction was stopped by the addition of 0.1 ml 100% (*w/v*) trichloroacetic acid, and the mixture was centrifuged at 1780 × *g* for 20 min. The amount of uric acid in the supernatant was determined by the absorbance at 292 nm against a blank and expressed as mIU/ml. A calibration curve was constructed using 10–50 mU/ml concentrations of standard XO solutions (Sigma-Aldrich). One unit of activity was defined as 1 mmol of uric acid formed per minute at 37 °C and pH 7.5.

MPO activity and MDA levels were measured using ELISA kits from Cusabio (Hubei, China) and Cayman Chemical Company (Ann Arbor, MI, USA), according to the manufacturer's instructions. MPO and MDA levels were determined from a standard curve and expressed in ng/ml. All assays were conducted in triplicate.

### SOD1, SOD2 and GPx analyses in spinal cord homogenates

SOD1 activity was measured by monitoring its capacity to inhibit the reduction of ferricytochrome *c* by xanthine/xanthine oxidase, as described by McCord and Fridovich.^51^ Protein samples were electrophoresed in 10% native polyacrylamide gels before SOD1 activity visualization, as described by Beauchamp and Fridovich.^52^ Briefly, the gel was soaked in 2.45 mM nitroblue tetrazolium solution for 15 min, followed by 30 min in 28 mM *N*,*N*,*N′′*,*N′′*-tetramethylethylene diamine and 28 *μ*M riboflavin in 0.36 mM potassium phosphate buffer (pH 7.8). The gel was then exposed to a fluorescent light source until the bands showed maximum resolution.

CAT activity was assayed at 25 °C by determining the rate of H_2_O_2_ degradation in 10 mM potassium phosphate buffer (pH 7.0), according to the method described by Aebi.^53^ An extinction coefficient of 43.6 mM/cm was used for the calculations. One unit was defined as the consumption of 1 pmol of H_2_O_2_ per min and the specific activity was reported in units/mg protein.

GPx activity was assayed by measuring nicotinamide adenine dinucleotide phosphate (NADPH) oxidation using *t*-butyl-hydroperoxide as a substrate, as described by Maral *et al.*^54^ Briefly, the reaction was carried out at 25 °C in 600 *μ*l of a solution containing 100 mM potassium phosphate buffer (pH 7.7), 1 mM EDTA, 0.4 mM sodium azide, 2 mM glutathione, 0.1 mM NADPH, 0.62 U of glutathione reductase and 50 *μ*l of homogenate.

### Immunohistochemistry for Iba-1 and HNE in the spinal cord

In brief, sections were incubated with a mouse anti-Iba-1 (1:50, Wako, Osaka, Japan) or a mouse anti-4-HNE (1:100, OXIS International, Portland, OR, USA) overnight at room temperature. Sections were incubated with FITC-conjugated anti-mouse IgG and mounted on gelatin-coated slides in antifade mounting medium (VECTASHIELD^®^, Vector). The immunoreactions were observed under the confocal microscope (LSM510 META NLO, Carl Zeiss, Göttingen, Germany).

### Statistical analysis

The data were expressed as the mean of the experiments performed for each experimental investigation. The differences among the means were statistically analyzed using a one-way analysis of variance, followed by Bonferroni’s *post hoc* test in order to compare the effectiveness of PEP-1-PDIA3 against ischemic damage.

## Figures and Tables

**Table 1:** Physiological parameters before and after ischemic surgery

	**pH**	**Distal MAP (mmHg)**	**PaCO**_**2**_ **(mmHg)**	**PaO**_**2**_ **(mmHg)**	**Glu (mM)**
*Pre-ischemia*					
Control	7.39±0.03	84.1±9.45	36.9±4.08	103.6±9.24	6.42±0.99
Vehicle	7.40±0.02	84.6±8.79	37.6±3.95	101.8±8.93	6.36±1.12
Tat-PDIA3 (0.5 mg/kg)	7.42±0.03	83.8±8.86	37.3±3.71	103.9±9.59	6.51±0.93
Tat-PDIA3 (1.5 mg/kg)	7.41±0.02	83.1±9.94	37.7±4.12	102.7±9.77	6.31±0.98
Tat-PDIA3 (4.5 mg/kg)	7.42±0.03	84.2±8.15	37.2±3.55	103.4±8.31	6.44±1.16
*Reperfusion 10 min*					
Control	7.41±0.04	85.9±9.12	36.6±3.88	104.1±8.94	6.52±1.18
Vehicle	7.37±0.06	89.4±11.6	38.2±4.61	109.6±10.3	6.89±1.37
Tat-PDIA3 (0.5 mg/kg)	7.39±0.04	88.1±9.94	39.2±5.14	111.7±12.8	7.03±1.31
Tat-PDIA3 (1.5 mg/kg)	7.38±0.05	85.6±10.2	37.9±3.95	115.2±11.0	7.25±1.54
Tat-PDIA3 (4.5 mg/kg)	7.36±0.09	92.7±12.4	40.4±7.14	103.2±13.2	6.94±1.40

**Table 2:** Summary of the protein properties and identification of proteins using MALDI.

**Proteins (gI accession number)**	**Number of peptides matched in the identified protein**	**Percentage covering of the matched peptides (%)**	**pI, Mr (kDa)**	**MOWSE**	**Fold increase/decrease(Δ)**
Heat shock protein HSP 90-alpha (338817950)	17/74	25	4.88, 80.082	75	Δ4.6
Protein disulfide-isomerase A3 precursor (284005547)	18/106	39	5.98, 56.671	97	Δ3.2
Glucose-6-phosphate 1-dehydrogenase (284005000)	17/102	38	6.35, 59.796	80	Δ3.4
Elongation factor 1-alpha 1 (56405012)	11/69	25	9.10, 50.451	57	Δ13.9
Phosphatidylethanolamine-binding protein 1 (75047560)	8/71	44	6.59, 21.095	60	Δ3.0
Myelin basic protein (17378880)	8/56	45	11.38, 18.206	58	3.2
Septin-8 (284004948)	21/119	50	5.73, 56.187	100	5.3
