# Supplementary figures and images for: Tat-protein disulfide-isomerase A3: a possible candidate for preventing ischemic damage in the spinal cord
Source: Cell Death Dis. 2017 Oct 5;8(10):e3075–. doi: 10.1038/cddis.2017.473 (PMC5680594; doi:10.1038/cddis.2017.473)

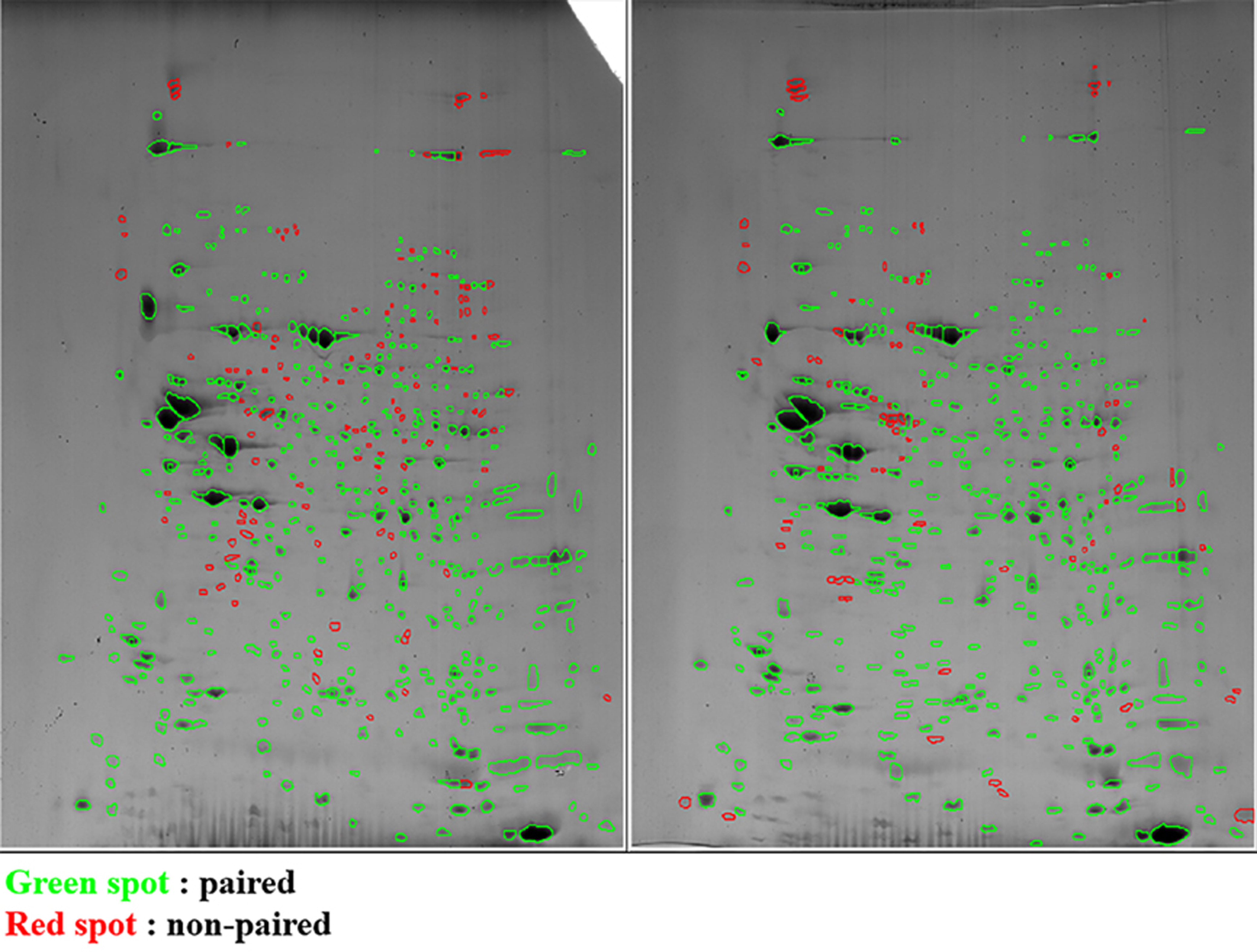

Supplement: Supplementary Figure 1 [file cddis2017473x1.tif]

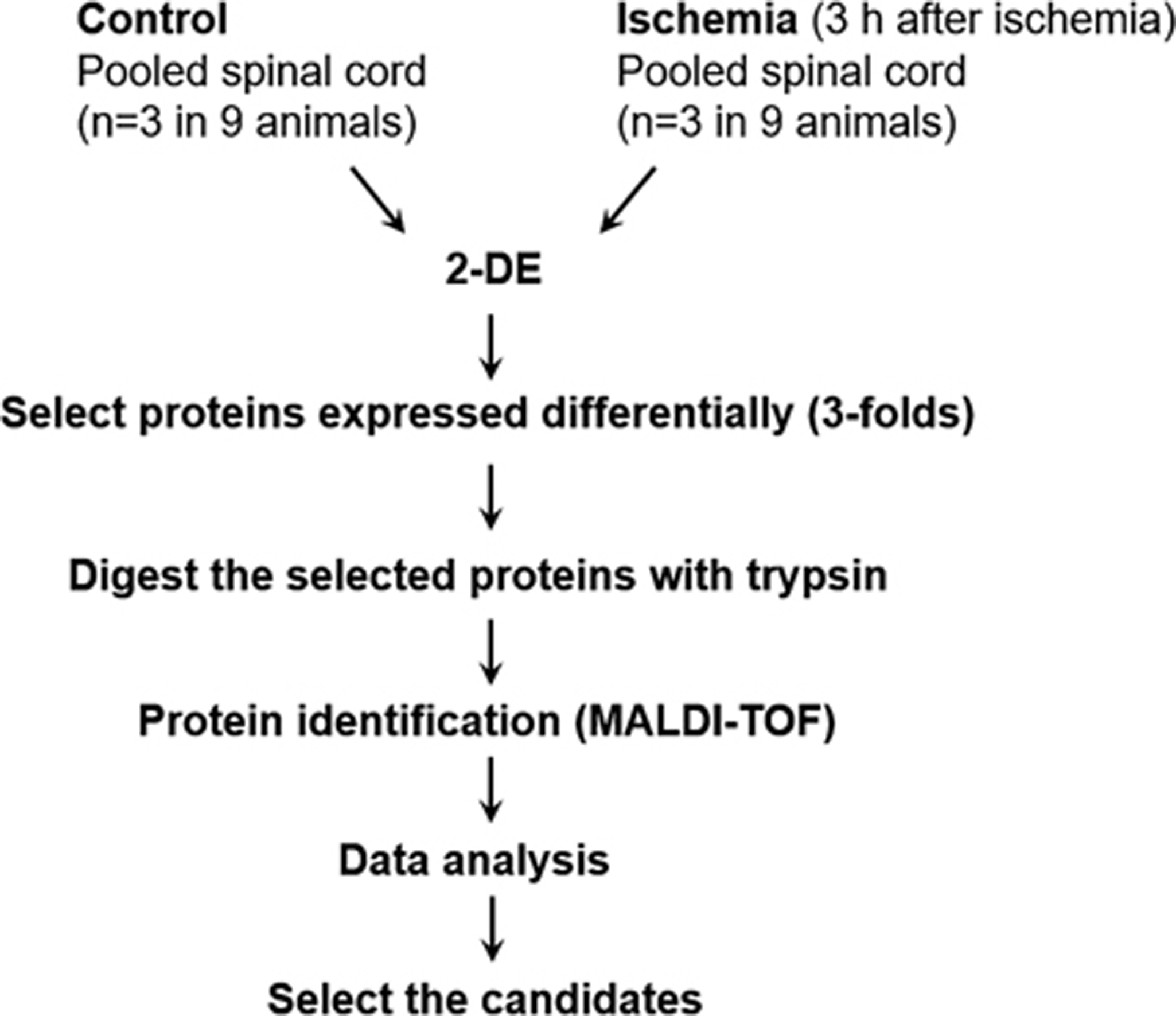

Supplement: Supplementary Figure 2 [file cddis2017473x2.tif]
