# Supplementary material for: Tat-protein disulfide-isomerase A3: a possible candidate for preventing ischemic damage in the spinal cord
Source: Cell Death Dis. 2017 Oct 5;8(10):e3075–. doi: 10.1038/cddis.2017.473 (PMC5680594; doi:10.1038/cddis.2017.473)
Supplement: Supplementary Figure Legends [file cddis2017473x3.doc]

**Legend for Supplementary Figures**

**Supplementary Figure 1** Two-dimensional gel electrophoresis (2DE) gel of spinal cord proteins to compare the proteomes of spinal cord proteins in the control and ischemia-operated group, 3 h after ischemia/reperfusion (A). Paired and non-paired spots are shown in green and red, respectively (B).

**Supplementary Figure 2** Schematic drawing of the overall workflow in proteomics study.
